# Acceptance of human papillomavirus vaccination and parents’ willingness to vaccinate their adolescents in Ethiopia: a systematic review and meta-analysis

**DOI:** 10.1186/s13027-023-00535-6

**Published:** 2023-10-11

**Authors:** Awoke Derbie, Daniel Mekonnen, Eyaya Misgan, Melanie Maier, Yimtubezinash Woldeamanuel, Tamrat Abebe

**Affiliations:** 1 Department of Medical Microbiology, College of Medicine and Health Sciences, Bahir Dar University, Bahir Dar, Ethiopia; 2 Centre for Innovative Drug Development and Therapeutic Trials for Africa (CDT-Africa), Addis Ababa University, Addis Ababa, Ethiopia; 3 Department of Health Biotechnology, Biotechnology Research Institute, Bahir Dar University, Bahir Dar, Ethiopia; 4 Department of Medical Microbiology, Immunology, and Parasitology, School of Medicine, College of Health Sciences, Addis Ababa University, Addis Ababa, Ethiopia; 5 Department of Gynecology and Obstetrics, College of Medicine and Health Sciences, Bahir Dar University, Bahir Dar, Ethiopia; 6 Department of Virology, Institute of Medical Microbiology and Virology, Leipzig University Hospital, Leipzig, Germany

**Keywords:** HPV, Vaccination uptake, Parents‘ willingness, Ethiopia

## Abstract

**Introduction:**

Despite the global vaccination campaign to prevent HPV-related morbidity, HPV vaccination uptake remains unacceptably low in the developing world, like Ethiopia. For strong interventional measures, compiled data in the field is required which is otherwise missed in the Ethiopian context. Therefore, this systematic review aimed to provide an estimate of the HPV vaccination uptake, mothers‘ willingness to vaccinate their adolescent girls, and associated factors in Ethiopia.

**Methods:**

Articles were systematically searched using comprehensive search strings from PubMed/Medline, SCOPUS, and grey literature from Google Scholar. Two reviewers assessed study eligibility, extracted data, and assessed the risk of bias independently. Meta-analysis was performed using STATA v 14 to pool the vaccination uptake and mothers‘ willingness toward HPV vaccination in Ethiopia.

**Results:**

We included 10 articles published between 2019 and 2022 covering reports of 3,388 adolescent girls and 2,741 parents. All the included articles had good methodological quality. The pooled estimate of the proportion of good knowledge about HPV vaccination and the agreement of girls to get the vaccine was 60% (95%CI: 59–62) and 65% (95%CI: 64–67), respectively. The pooled estimate of vaccination uptake of at least one dose of HPV vaccine among girls was 55% (95%CI: 53–57). Positive attitudes to the vaccine, higher maternal education, and having knowledge about HPV and its vaccine were reported as statistically significant predictors. On the contrary, not having adequate information about the vaccine and concerns about possible side effects were reported as reasons to reject the vaccine. Likewise, the pooled estimate of mothers who were knowledgeable about HPV vaccination, who had a positive attitude, and willing to vaccinate their children were 38% (95%CI: 36–40) 58% (95%CI: 56–60), and 74% (95%CI: 72–75), respectively.

**Conclusions:**

Knowledge about the HPV vaccine among girls and their vaccination uptake is suboptimal that falls short of the 2030 WHO targets. Therefore, stakeholders need major efforts in rolling out vaccination programs and monitoring their uptake. Social mobilization towards primary prevention of HPV infection should focus on adolescents. The existing strategies need to address the predictors of uptake by educating girls and parents.

## Background

The prevalence of cervical cancer (CC) is steadily increasing in low-income countries and causes significant morbidity and mortality. Cervical cancer is one of the emerging public health challenges in Ethiopia. The incidence and prevalence are increasing from time to time [1]. According to the International Agency for Research on Cancer assessments, the estimated number of new CC cases at 7,500 in 2020 could intensify to 15,300 in 2040. Similarly, the mortality from the disease could increase from ~ 5,340 in 2020 to 11,000 in 2040 in Ethiopia [2]. Despite all these impacts, so far, the country does not introduce better CC screening practices and well-established CC vaccination programs. The visual inspection (VIA)-based screening coverage is insignificant (3.3%) among women aged 18–69 years [3–5].

The global scale-up HPV vaccination, HPV-based screening, and treatment of precancerous lesions are the recommended interventions to curb the burden of CC. The World Health Organization (WHO) has set a vision of a world where CC is eliminated as a public health problem using the life-course approach. By 2030, each country should meet the 90-70-90 targets; i.e. (1) 90% of girls shall be fully vaccinated with HPV by age 15 years, (2) 70% of women shall be screened with a high-performance test by 35 years of age, and again by 45 years, and (3) 90% of women identified with cervical disease shall receive treatment [6].

Though Ethiopia has issued a guideline to meet the global targets, the life-course approach for CC prevention is at an early stage [5]. Most women are diagnosed at an advanced stage of the disease [7] due to a lack of an efficient program and low uptake of the available strategy [8]. The country launched the quadrivalent vaccination for the first time, with the support of the Global Alliance for Vaccine and Immunization (GAVI) in 2018.

Because HPV infection is transmitted sexually, the quadrivalent recombinant HPV vaccination strategy targets female adolescents aged 9 to 14 years, for whom the first vaccination dose should be administered before a sexual encounter. Despite the global vaccination campaign to prevent HPV-related morbidity, HPV vaccination uptake remains unacceptably low. The uptake of vaccination of young girls is low in low- and middle-income settings (13%) [9].

Since the vaccination program targets girls aged 9–14, the success of vaccination depends on the parental decision and their willingness to vaccinate their daughters. The involvement of parents in the decision to take the HPV vaccine for their children is very crucial for the acceptability and utilization of the vaccine [10]. Generally, the low HPV vaccination uptake is multifaceted and can be attributed in part to a variety of predictors such as low vaccine knowledge, vaccine side effects, and school attendance status. Besides, some parents’ beliefs that HPV vaccination might encourage promiscuity, earlier sexual debut in young girls, and that the vaccine might lead to unsafe sexual behavior were barriers to HPV vaccination uptake [11]. Additional barriers include parents’ knowledge, perceptions, and attitudes toward the HPV disease and the vaccine, along with the convenience of receiving the vaccine [12].

A systematically compiled nationwide data on HPV vaccination uptake in Ethiopia is missing which is otherwise important for strong interventional measures. Therefore, the current study was conducted to assess HPV vaccination uptake among female adolescents and to describe the knowledge and willingness of the HPV vaccine and associated factors among parents of female adolescents in Ethiopia.

### Review question

This systematic review addresses the following questions;

- What percentage of Ethiopian adolescent girls received the HPV vaccine?
- How much do parents know about vaccines and how willing were they to vaccinate their kids?

### Objective

The aim of this review was to describe the HPV vaccine uptake among female adolescents and to assess the knowledge and willingness of parents to vaccinate their adolescent girls.

## Methods

### Eligibility criteria

Studies were selected based on the following criterion: *Study design*: descriptive studies that reported the HPV vaccine uptake among female adolescents and the knowledge of women on this vaccination. *Participants*: female adolescent girls and parents of children. *Setting*: we included studies with the outcome of interest reported in Ethiopia. *Language and publication*: We included peer-reviewed published articles and unpublished preprints written in the English language.

### Information sources and search strategy

This review was done following the PRISMA guideline (Supplementary file 1). A computerized systematic strategy was adopted to search for articles in PubMed/Medline and SCOPUS. The last search was conducted on 20 April 2023. A manual search from Google Scholar and Google databases was also carried out for grey literature. The search terms were developed in line with the Medical Subject Headings (MeSH) thesaurus using a combination of key terms which are derived from the research question. The reference lists of retrieved articles were probed (forward and backward searching) to identify articles that were not retrieved from the databases’ manual search. The first two authors; AD and DM searched the articles independently.

The domains of the search terms were: ‘HPV vaccination’, ‘vaccination for cervical cancer’, ‘female adolescents’, ‘vaccination uptake’ ‘parental knowledge’, ‘parental willingness‘, and ‘Ethiopia’. We combined these terms using the Boolean operator “OR”, and “AND” accordingly. The full search strategy for the two databases is annexed in Supplement 1.

### Study selection

Studies that reported the HPV vaccine uptake among female adolescents and the knowledge and willingness of parents to vaccinate their children were included regardless of their year of publication. Searched articles were directly imported and handled using EndNote X9 citation manager (Thomson Reuters, New York, USA). Based on the PRISMA procedure, duplicated articles were excluded and the titles and abstracts of the remaining papers were screened sequentially for inclusion in full-text evaluation by the first two authors. Differences between the reviewers were resolved through discussion.

### Data collection process and data items

The extracted data items include the name of the first author, publication year, age range/mean of the study participants, sample size, variables related to the HPV vaccine uptake, and the knowledge and willingness of parents about the vaccination. The data were extracted from the included articles using piloted Excel data extraction sheet developed by the first author.

### Methodological quality appraisal of the included studies

The validity and methodological quality of all included studies were assessed using the Joanna Briggs Institute Critical Appraisal Checklist for prevalence data (Supplementary file 3). The tool consists of nine criteria that were checked as ‘yes’, ‘no’, ‘unclear’, or ‘not applicable’. After carefully evaluating the included articles against each criterion, studies were finally classified into three groups; a study that fulfilled > 80% of the criteria were considered as ‘good quality’. Similarly, a study that scored 50–80% and < 50% were rated as ‘fair’ and ‘poor’ quality, respectively. Fortunately, all the included studies scored > 80 and were judged as methodologically good.

### Data synthesis

Descriptive statistics, such as simple counts, ranges, and percentages were used to present the synthesized data. A systematic narrative synthesis was provided in which summary results were presented using text and tables. To pool the overall vaccination uptake and mother’s knowledge, meta-analysis was performed using STATA v 14 (Stata Corp. College Station, TX, USA) using a random effect model. Predictors were presented in the description. A predictor is considered for inclusion when two or more articles reported it as a statistically significant factor. The heterogeneity of the included studies was assessed using the *I*^2^ test and *I*^2^ = > 50% was considered as high heterogeneity among the results of the included studies. Additionally, to assess the presence of publication bias, a funnel plot was performed. To assess the influence of individual studies on the pooled data, a sensitivity analysis was performed.

### Operational definition

#### Vaccination uptake

the proportion of adolescent girls who have received the HPV vaccine.

#### Knowledge

the included studies used a series of different items to measure adolescent girls and mothers‘ knowledge of HPV vaccination. The level of knowledge was measured using items related to risk factors for cervical cancer, the benefit of the vaccine, their attitude, and the prevention methods of cervical cancer. Studies used the cumulative mean score of the participants about cervical cancer to measure their knowledge. Based on this, they labeled poor knowledge for those who had scored less than the mean and good knowledge for those who had scored greater than or equal to the mean value.

## Results

### Search results

From the computerized systematically searched databases and other sources, a total of 44 articles were retrieved and sequentially screened for inclusion in the analysis using the PRISMA flow chart (Supplementary file 2). Ten articles met our inclusion criteria and were included in the systematic review and meta-analysis.

### Characteristics of the included studies

The characteristic of the included studies is summarized in Table 1. All studies were published in the period of 2019 and 2022 and used a cross-sectional study design to describe the vaccination uptake and willingness of parents to vaccinate their adolescent girls. These articles used a questionnaire adapted from previously published similar articles to generate data.

The included studies [10–19] were reported from the three regions of the country; Amhara, Oromia, and Southern Nations, Nationalities, and Peoples Regions (SNNP). The number of participants in each included article varied from 414 to 899. Overall, this review contains reports of 3,388 adolescent girls and 2,741 parents. The reported age group and the mean age of the participants were variable. The age group of the adolescent girls was 9–18 years and the mean age of the parents varied between 28.6 and 40.9 years (Table 1).

**Table 1:** Descriptive summary of studies included in the systematic review

Author, year	Study area	Study participants		
		**Sample size**	**Students’ age group /mean mother’s age**	**Students’ grade**
Ukumo, 2020	Arba Minch	516	9–14	NR
Mihretie, 2020	Debre Tabor	824	13–19	7–10
Beyene, 2020	Ambo	414	14–18	7–12
Kassa, 2020	Minjar Shenkora	591	11–15	5–8
Likneh, 2021	Bahir Dar	620	15–24	11–12
Abera, 2022	Nekemtie	423	14–18	9–12
Alene, 2019	Gonder	899	39	NA
Destaw, 2021	Bench-Shekor Zone	502	28.6	
Humnesa, 2021	Meta Robi (W.Showa)	619	35.1	
Mihret, 2022	Debre Tabor	721	40.9	

W: West, NA: not applicable, NR: Not reported

### HPV vaccination uptake

**Fig. 1:**
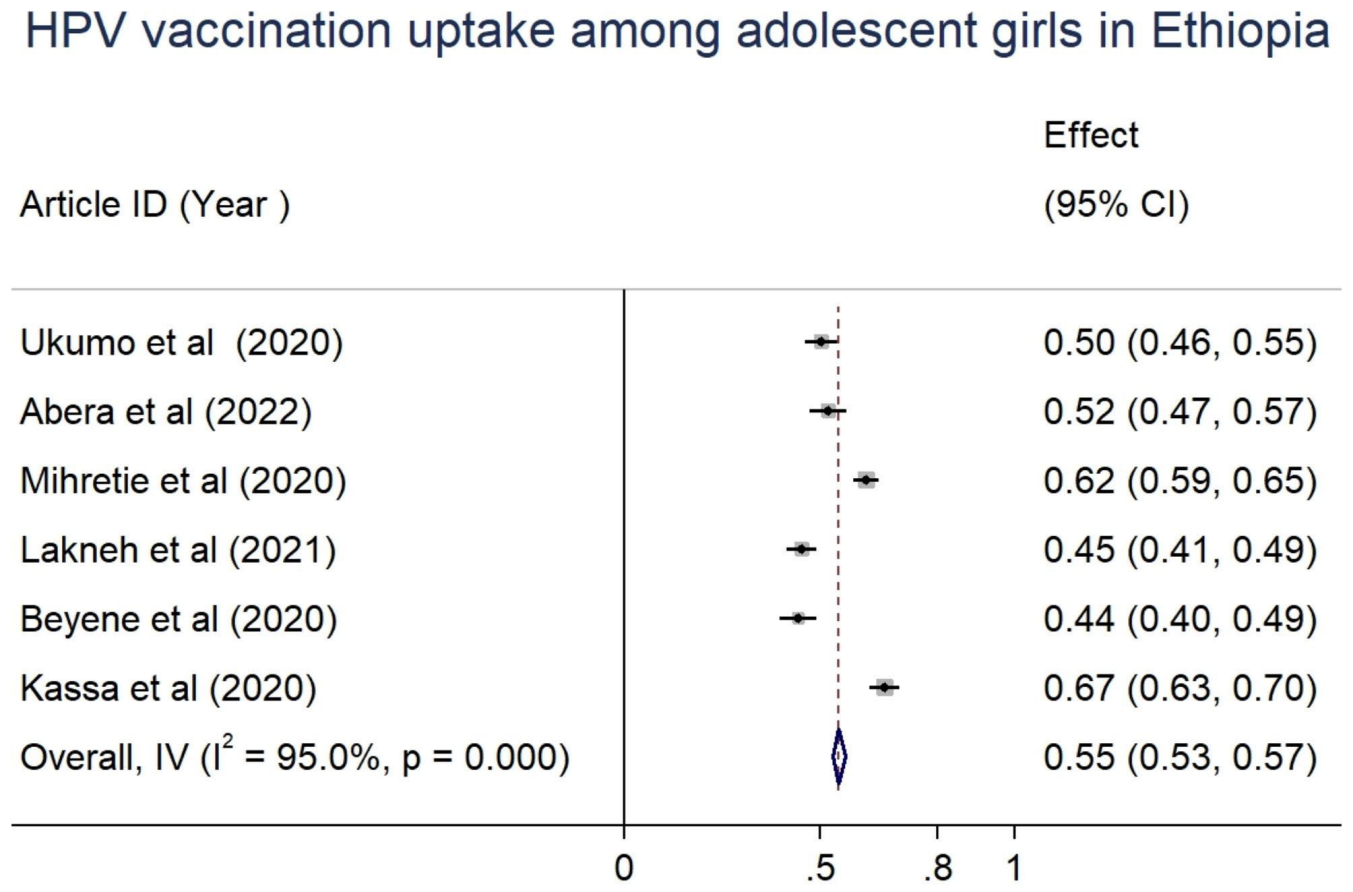
Forest plot showing the pooled estimate of HPV vaccination uptake among adolescent girls in Ethiopia, 2020-22

The pooled estimate of the proportion of good knowledge about HPV vaccination and the agreement of adolescent girls to get the vaccine were at 60% (95%CI: 59–62) and 65% (95%CI: 64–67), respectively. The included studies reported the proportion of vaccination uptake between 44.1% [12] and 66.5% [13]. The pooled estimate of vaccination uptake of at least one dose of HPV vaccine among adolescents in Ethiopia was 55% (95%CI: 53–57) (Table 2; Fig. 1).

**Table 2:** The proportion of good knowledge about HPV vaccination and the agreement of adolescent girls to get the vaccine in Ethiopia

Author, year	Agreed to be vaccinated	Good knowledge about the vaccine	Vaccination uptake
Ukumo, 2020	318 (61.6%)	388 (75.2)	260(50.4%)
Mihretie, 2020	542(65.8%)	488 (59.2%)	542(61.9%)
Beyene, 2020	273 (74.6%)	193 (52.7%)	184(44.4%)
Kassa, 2020	296 (50.1%)	300 (50.8%)	393 (66.5%)
Likneh, 2021	478(77%)	360 (58.1%)	281(45.3%)
Abera, 2022	239 (56.5%)	264 (62.4%)	220 (52%)
Pooled estimates (95%CI), I^2^	**65% (64–67), 96.3%**	**60%(59–62), 94.7%**	**55% (53–57), 95%**

### Factors associated with HPV vaccination uptake

The statistically significant predictors for the uptake of the HPV vaccine by adolescent girls are indicated in Table 3. Positive attitudes to the vaccine, higher maternal education, and having knowledge about HPV and its vaccine were reported by two or more articles as important statistically significant predictors. On the contrary, not having adequate information about the vaccine and concerns about possible side effects were reported as reasons for not taking the vaccine.

**Table 3:** Factors associated with HPV vaccination uptake among adolescent girls in Ethiopia

Author, year	Predictors to get the vaccination	Reason for not talking about the vaccine
Ukumo, 2020	o Girl’s age > 14, o Mother’s education secondary and above, o Childhood vaccination history, o Positive attitude to accept HPV vaccination, o Having awareness	NR*
Mihretie, 2020	o Discussion of reproductive health issues with family, o Having good knowledge about the HPV vaccine, o Positive attitude toward the HPV vaccine	• The vaccine might cause disease, • Fear of needle injection, • Perceived that the vaccine might cause other cancer
Beyene, 2020	o Hearing about HPV vaccine, o Having awareness about the disease, o Favorable attitude to the vaccine	• Not having information, • Fear of side effects, • Less confidence on its benefit, • Absence from class at the time of vaccination, • Negative attitude
Kassa, 2020	o Urban residence, o Good knowledge and attitude	• Worried about the vaccine, • Do not know where to get the vaccine, • Not informed by health care, • Believe no need for vaccine
Likneh, 2021	o Participants who discussed reproductive health issues, o Well-informed about the HPV vaccine	NR
Abera, 2022	o Having good knowledge of HPV and its vaccine, o Having a positive attitude towards HPV vaccination, o Higher maternal education level (college and above), o Urban residence	• Lack of information about the HPV vaccine, • Needle phobia, • Concerns about possible side effects of the vaccine, • Didn’t know where to get the vaccine

NR*: Not Reported

#### Mothers‘ willingness to vaccinate their children

**Fig. 2:**
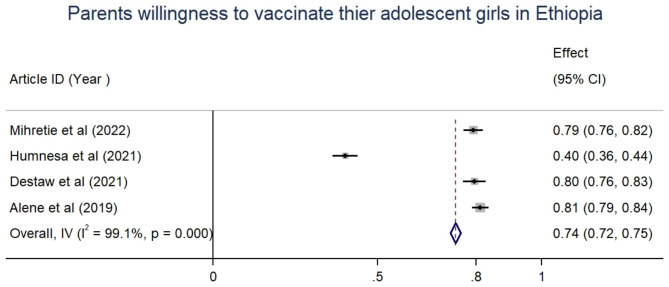
Forest plot showing the pooled prevalence of mothers willingness to vaccinate thier adolescent girls in Ethiopia, 2019-22

Four studies reported mothers‘ willingness to vaccinate their children. The pooled estimate of mothers who were knowledgeable about HPV vaccination and those who had a positive attitude to vaccinating their children were 38% (95%CI: 36–40) and 58%(95%CI: 56–60), respectively (Table 4). The proportion of mothers who were willing to vaccinate their adolescent girls was 40.2% [18] to 81.3% [16] with a pooled estimate of 74% (95%CI: 72–75) (Fig. 2).

**Table 4:** Mothers‘ knowledge, positive attitude, and willingness to vaccinate their adolescent girls in Ethiopia

Author, year	Mothers‘ Knowledge about HPV vaccination and CC	Positive attitude toward HPV vaccination	Willingness to vaccinate their children
Alene, 2019	335(37.3)	538 (59.9%)	731 (81.3%)
Mihretie, 2020	332 (46%)	443(61.4%)	570(79.1%)
Destaw, 2021	142 (28.3%)	348 (69.3)	399 (79.5%)
Humnesa, 2021	242 (39.1%)	249(40.2)	249 (40.2%)
Pooled estimates (95%CI), I^2^	**38% (36–40), 92.9%**	**58%(56–60),97.4%**	**74%(72–75), 99.1%**

### Factors associated with mothers‘ willingness to vaccinate their children

Parents having media exposure, having good knowledge about HPV infection, and positive attitude towards HPV vaccination were reported to be important predictors to vaccinate their adolescent girls (Table 5).

**Table 5:** Factors associated with mothers‘ willingness to vaccinate their children in Ethiopia

Author, year	Predictors to vaccinate their girl
Alene, 2019	o Being from the richest household, o Good knowledge of CC, o Positive attitude towards HPV vaccination
Mihretie, 2020	o Parents having media exposure, o Had good knowledge of HPV infection, and HPV vaccine, o Positive attitude, and positive perceived behavioral control toward the HPV vaccine
Destaw, 2021	o Primary education and above, o Having good knowledge, o Positive attitude
Humnesa, 2021	o Age less than 30 years, o Urban residents, o Rich household

## Discussion

The global scale-up of HPV vaccination, HPV-based screening, and treatment of precancerous lesions are the recommended interventions to curb the burden of cervical cancer worldwide. This particular review summarized the findings of articles containing information about the level of adolesent girls HPV vaccination uptake and mothers willingness to vaccinate thier children in Ethiopia.

In our review, the included studies reported the proportion of vaccination uptake between 44.1% [12] and 66.5% [13]. The pooled estimate of vaccination uptake of at least one dose of HPV vaccine among adolescents in Ethiopia was 55% (95%CI: 53–57), which is quite low and represents the wide gap that needs to be bridged before achieving the WHO strategy of having 90% of girls fully vaccinated by the age of 15 years in 2030 [6]. Our finding suggests that a significant proportion of adolescent girls remain largely unprotected [20, 21]. According to the WHO recommendation, the strategy to eliminate cervical cancer requires fully vaccinating 90% of girls by 15 years of age, screening 70% of women with a high-performance test by 35 years of age and again at 45 years of age, and 90% of women identified with cervical disease receiving treatment [6].

If HPV vaccination and cervical screening are scaled up, the annual cervical cancer incidence can be brought down by significant levels. HPV-based vaccination in Ethiopia was started in October 2018 for school girls. The available vaccine in the country is Gardasil-4, which targets HPV6, 11, 16, and 18 [22]. According to the information from WHO African region report, so far about 2 million girls aged 9–14 were vaccinated in the country [23]. The HPV vaccination is not part of the national immunization program in Ethiopia partly because of the cost of the vaccine. However, our review result showed that the uptake was low even if the vaccine was made available for the public for free. Using Gardasil-4, that doest target the most high-risk HPVs in Ethiopia [22], coupled with low vaccine acceptance will complicate the fight against CC in Ethiopia.

The level of HPV vaccination uptake in different African countries was also reported to be low. For example, a study in Nigeria showed that the level of knowledge of adolescent girls on HPV vaccines was low and the vaccination uptake was 2.1-4%. The most common reason given for not having taken the vaccine was unawareness of the vaccine [24, 25]. Similarly, a study in Uganda and Kenya showed that the vaccination uptake of adolescent girls was 17.61% [26] and 33% [27], respectively. Further, in a similar meta-analysis study on HPV vaccination uptake in low and middle-income countries, the pooled estimate of vaccination uptake of any dose was about 61% with a wide range of percentages of uptake reported from various countries in the period of 2006 to 2020 [28].

Another systematic review on the uptake of HPV vaccination among adolescent girls showed that the vaccination uptake rate for at least one dose varied significantly among countries, ranging from 2.4% in Hong Kong to 94.4% in Scotland [29]. Similarly, the HPV vaccination uptake of female students (age 9–12 years) from 31 primary schools in South Africa was reported to be 98% [30]. More than 90% HPV vaccination uptake was also reported among girls (9–15 years) in the Europian union [31].

The difference in the reports of the HPV vaccination uptake across these studies might be due to disparities in socioeconomic status, health information access, and tools used to measure the acceptance of HPV vaccination. Specifically, the higher vaccination uptake in the developed world might be because girls might have good accessibility to the HPV vaccine with all the necessary information.

The acceptance of the HPV vaccination in our review is low which might be due to poor health information access, less involvement of girls participating in health-related school clubs, and sociocultural influence on the female gender. In our review, the pooled estimate of the proportion of good knowledge about HPV vaccination and the agreement of adolescent girls to get the vaccine were 60% and 65%, respectively. These figures might be directly related to the low uptake of the vaccine. A review article in Sub-Saharan Africa also showed that there was a relatively higher level of willingness to get the HPV vaccine, but low levels of knowledge and awareness about the HPV vaccine was reported [32].

The influence of knowledge and perceptions of HPV vaccination suggests the importance of tailored health education on HPV immunization. Therefore, actions aimed at creating a positive attitude toward the HPV vaccine, sensitization of adolescents about the vaccine, and conducting community outreaches are timely areas of interventions that the stakeholders should consider. As social media outlets are becoming an increasing source of information in Ethiopia, using these platforms HPV related information, attitudes, and behavioral-related information can be easily made available to the people at large.

The United States, Australia, and Canada were the first countries to implement HPV vaccination as part of their national immunization programs since 2006 [24]. In contrast, most low- and middle-income countries started the HPV vaccination in 2018/19. Only about 31% of countries in the WHO AFRO region had begun vaccination as part of their national immunization program [24]. By the middle of 2020, 15 to 20% of adolescent girls took at least one dosage globally in terms of coverage. As a result, many girls who reside in nations that have not yet incorporated the HPV vaccine into their national immunization schedules are unprotected [20–24].

Our review also identified factors associated with HPV vaccination practice among adolescent girls. The included studies in our review reported different statistically significant predictors for the uptake of the HPV vaccine by girls. Positive attitudes to the vaccine, higher maternal education, and having knowledge about HPV and its vaccine were reported by two or more articles as important predictors to take the vaccine. On the contrary, not having adequate information about the vaccine and concerns about possible side effects were reported as reasons for not taking the vaccine. A similar study in Nigeria reported that The most common reason given for not having taken the vaccine was unawareness about the HPV vaccine (98%) [24, 25].

Proper strategies to overcome these barriers are needed to ensure successful vaccination uptake. There is no single solution to increase vaccination uptake and different approaches may be better suited to certain populations. Hence, it is suggested that barriers to the uptake of the vaccine should be addressed, and that school-based sexual health education of HPV infection and vaccine promotion should be practiced in Ethiopia [29]. A study in Nigeria reported that the perception of susceptibility to HPV infection by girls was significantly associated with acceptance of the vaccines [33].

A similar study in Latin America indicated that there were several reasons attributed to low vaccination uptake among adolescent girls: limited knowledge of HPV and HPV vaccine, misguided safety concerns, high cost, cultural barriers, and the COVID-19 pandemic [34]. There is an urgent need for more education to inform the public about HPV, cervical cancer, and the HPV vaccine, particularly to key parties, (adolescents and their parents), to leverage high levels of willingness and acceptability of the HPV vaccine toward successful implementation of HPV vaccination program in Ethiopia [35]. It is suggested that to increase the HPV vaccination uptake, strategies targeting adolescents/parents focussed on reminder-based regular announcements, education, information, and communication activities, and multicomponent approaches [35].

With regard to mothers‘ willingness to vaccinate their children, the pooled estimate of mothers who were willing to vaccinate their adolescent girls was 74%. Similar studies in Nigeria reported that 70-79.2% of mothers of adolescent girls demonstrated a willingness to vaccinate their daughters against HPV. Those mothers who were unwilling to vaccinate their adolescent girls reason out that it may encourage sexual promiscuity [33]. Likewise, parental HPV vaccine acceptance in Indonesia was 96.1%, which is higher than our report which may be because of the difference in socio-economic status of these people. But, the study demonstrated that knowledge about HPV and cervical cancer is low [36].

In our study, the pooled estimate of mothers who were knowledgeable about HPV vaccination and those who had a positive attitude to vaccinate their children were 38% and 58%, respectively. Similarly, in Nigeria, only 19.0% of mothers had good knowledge about cervical cancer prevention strategies [25]. Further, Jaspers et al., from Indonesia demonstrated that knowledge about HPV and cervical cancer is low among mothers of adolescent girls [36]. While the involvement of parents in the decision of their children to take the HPV vaccine [20, 21], the reported quite low knowledge and attitude towards vaccine need mitigating action.

For the HPV vaccination uptake barriers include parents‘ knowledge, perceptions, and attitudes toward the HPV infection [21]. In our review, parents having media exposure, having good knowledge about HPV infection, and positive attitude towards HPV vaccination were reported to be important predictors to vaccinate their adolescent girls. In contrast, a study reported about mothers‘ perceived reasons why they failed to vaccinate their girl and the important factors identified were the absence of information about HPV, their belief that the vaccine may affect their child‘s fertility, fear of side effects, and fear of needle injection [11]. A similar survey conducted to identify trends in the main reasons of parents of unvaccinated children in the United States showed that the top five most frequently cited reasons for not intending to vaccinate their children included “not necessary,” “safety concerns,” “lack of recommendation,” “lack of knowledge,” and “not sexually active.” Overall, parental HPV vaccine hesitancy decreased by 5.5% annually for some years [37]. Over time, more parents stated that they did not intend to vaccinate their adolescent children against HPV due to concerns about the vaccine safety [37].

It is reported that health education intervention was found to be effective at improving the parental willingness to vaccinate their adolescents with the HPV vaccine. A multipronged approach in educating the parents of adolescents about the benefit of the HPV vaccine in reducing and preventing the infection and its effects. Educated mothers might feel confident to vaccinate their daughters against HPV by retrieving medical information on the risks of contracting HPV infections [15].

### Strength and limitations

To the best of our knowledge, this systematic review reported the latest summarized finding of HPV vaccination uptake and parental willingness to vaccinate adolescent girls in the Ethiopian context. However, our findings should be interpreted with caution due to some drawbacks; the studies were reported from only some regions of the country. The absence of data from the rest of the regions in the country might compromise our conclusion. The other snare of this review is the presence of high heterogeneity between the included articles.

## Conclusions

In this study, the level of HPV vaccination practice among adolescent girls in Ethiopia was 55%. The pooled estimate of mothers who were willing to vaccinate their adolescent girls was 74%. The current levels of vaccination in the population of adolescent girls fall short of the 2030 WHO targets. Therefore, stakeholders need major efforts in rolling out vaccination programs and monitoring their uptake. Social mobilization towards primary prevention of HPV infection should focus on adolescents and mothers. Community education on cervical cancer and its prevention to increase awareness is necessary. Moreover, efforts should be made to enhance awareness about HPV vaccination through mass and social media outlets and other health education means towards HPV vaccination. School-based clubs should be considered to provide adolescent-friendly information to create basic awareness and as a result to improve the observed low level of HPV vaccination uptake in Ethiopia.

## Data Availability

The original data source could be shared upon the request of the principal investigator.

## References

[1] World Health Organization. Global strategy to accelerate the elimination of cervical cancer as a public health problem, 2020. Available at: [https://apps.who.int/iris/bitstream/handle/10665/336583/9789240014107-eng.pdf].

[2] World Health Organization. International Agency for Research on Cancer. Estimated number of cervical cancer incidence and mortality in Ethiopia. 2023. Available at: https://gco.iarc.fr/tomorrow/en/dataviz/isotype?cancers=23&single_unit=500&populations=231&group_populations=1&multiple_populations=1&sexes=0&types=1&age_start=0.

[3] Bruni L, Albero G, Serrano B, ICO/IARC Information Centre on HPV and Cancer (HPV Information Centre). Human Papillomavirus and Related Diseases in the World. Summary Report 2023. Available at: https://hpvcentre.net/statistics/reports/XWX.pdf.

[4] Ethiopian Public Health Institute. Ethiopia mini demographic and health survey. 2019. Available at: https://ephi.gov.et/wp-content/uploads/2021/05/Final-Mini-DHS-report-FR363.pdf.

[5] Federal Democratic Republic of Ethiopia MoH: Guideline For Cervical Cancer Prevention And Control In Ethiopia. 2021. Available at: https://www.iccp-portal.org/system/files/plans/Guideline%20Eth%20Final.pdf.

[6] WHO. Global strategy to accelerate the elimination of cervical cancer as a public health problem. Geneva [https://www.who.int/publications/i/item/9789240014107].

[7] McGuire JK, Kabagenyi F, Peer S (2023). Human papillomavirus vaccination in Africa: an airway perspective. Int J Pediatr Otorhinolaryngol.

[8] Derbie A, Mekonnen D, Nibret E (2023). Cervical cancer in Ethiopia: a review of the literature. Cancer Causes Control.

[9] Bruni L, Diaz M, Barrionuevo-Rosas L (2016). Global estimates of human papillomavirus vaccination coverage by region and income level: a pooled analysis. Lancet Glob Health.

[10] Ukumo EY, Weldehawariat FG, Dessalegn SA, Minamo DM, Weldehawaryat HN. Acceptance of Human Papillomavirus Vaccination and Associated Factors among Girls in Arba Minch Town, Southern Ethiopia, 2020. Infectious diseases in obstetrics and gynecology. 2022:7303801. 10.1155/2022/7303801 PMC9750771 36531338

[11] Mihretie GN, Liyeh TM, Ayele AD, Belay HG, Yimer TS, Miskr AD, Kassa BG, Tefera AG, Dagnaw E, Belachew YY (2023). Female adolescents’ knowledge and acceptability of human papillomavirus vaccine in Debre Tabor Town, Ethiopia: a cross-sectional study. BMJ Open.

[12] Beyen MW, Bulto GA, Chaka EE, Debelo BT, Roga EY, Wakgari N, Danusa KT, Fekene DB (2022). Human papillomavirus vaccination uptake and its associated factors among adolescent school girls in Ambo town, Oromia region, Ethiopia, 2020. PLoS ONE.

[13] Kassa HN, Bilchut AH, Mekuria AD, Lewetie EM (2021). Practice and Associated factors of human papillomavirus vaccination among Primary School students in Minjar-Shenkora District, North Shoa Zone, Amhara Regional State, Ethiopia, 2020. Cancer Manage Res.

[14] Lakneh EA, Mersha EA, Asresie MB, Belay HG (2022). Knowledge, attitude, and uptake of human papillomavirus vaccine and associated factors among female preparatory school students in Bahir Dar City, Amhara Region, Ethiopia. PLoS ONE.

[15] Abera M, Tiliksew Ayalew T, Mengesha. Human Papillomavirus vaccination practice and its associated factors among secondary school female students in Nekemte town, Oromia region, Ethiopia, 2022.

[16] Alene T, Atnafu A, Mekonnen ZA, Minyihun A (2020). Acceptance of Human Papillomavirus Vaccination and Associated factors among parents of daughters in Gondar Town, Northwest Ethiopia. Cancer Manage Res.

[17] Destaw A, Yosef T, Bogale B (2021). Parents willingness to vaccinate their daughter against human papillomavirus and its associated factors in bench-sheko zone, southwest Ethiopia. Heliyon.

[18] Humnesa H, Aboma M, Dida N, Abebe M. Knowledge and attitude regarding human papillomavirus vaccine and its associated factors among parents of daughters age between 9–14 years in central Ethiopia, 2021. J Public Health Afr 2022, 13(3). 10.4081/jphia.2022.2129 PMC9614691 36313923

[19] Mihret G, Anteneh TA, Abiy SA, Bewota MA, Aynalem GL (2023). Parents’ willingness to vaccinate their daughters with human papillomavirus vaccine and associated factors in Debretabor town, Northwest Ethiopia: a community-based cross-sectional study. Hum Vaccines Immunotherapeutics.

[20] Aruho C, Mugambe S, Baluku JB, Taremwa IM (2022). Human papillomavirus vaccination uptake and its predictors among female adolescents in Gulu Municipality, Northern Uganda. Adolesc Health Med Ther.

[21] Bartlett JA, Peterson JA (2011). The uptake of human papillomavirus (HPV) vaccine among adolescent females in the United States: a review of the literature. J School Nursing: Official Publication Natl Association School Nurses.

[22] Derbie A, Mekonnen D, Nibret E, Maier M, Woldeamanuel Y, Abebe T (2022). Human papillomavirus genotype distribution in Ethiopia: an updated systematic review. Virol J.

[23] Ethiopia immunizes over 2 million girls against human papillomavirus (HPV). [https://www.afro.who.int/news/ethiopia-immunizes-over-2-million-girls-against-human-papillomavirus-hpv].

[24] Oboro IL, Ogaji DS (2023). Knowledge and uptake of human papillomavirus vaccine among female adolescents in Port Harcourt. A call for urgent intervention. Int STD Res Reviews.

[25] Haleemat WA, Oluchi JK-O, Ifeoma PO, Kofoworola AO. Parental willingness to vaccinate adolescent daughters against human papillomavirus for cervical cancer prevention in Western Nigeria. PAMJ. 2020;36(112). 10.11604/pamj.2020.36.112.19007 PMC7406451 32821323

[26] Kisaakye E, Namakula J, Kihembo C, Kisakye A, Nsubuga P, Babirye JN (2018). Level and factors associated with uptake of human papillomavirus infection vaccine among female adolescents in Lira District, Uganda. Pan Afr Med J.

[27] Karanja-Chege CM (2022). HPV Vaccination in Kenya: the Challenges faced and strategies to increase uptake. Front Public Health.

[28] Dorji T, Nopsopon T, Tamang ST, Pongpirul K. Human papillomavirus vaccination uptake in low-and middle-income countries: a meta-analysis. eClinicalMedicine 2021, 34. 10.1016/j.eclinm.2021.100836 PMC8102703 33997733

[29] Loke AY, Kwan ML, Wong YT, Wong AK (2017). The Uptake of Human Papillomavirus Vaccination and its Associated factors among adolescents: a systematic review. J Prim care Community Health.

[30] Moodley I, Tathiah N, Mubaiwa V, Denny L. High uptake of Gardasil vaccine among 9–12-year-old schoolgirls participating in an HPV vaccination demonstration project in KwaZulu-Natal, South Africa. South African medical journal = Suid-Afrikaanse tydskrif vir geneeskunde. 2013;103(5):318–21. 10.7196/samj.6414 23971122

[31] Nguyen-Hu NH, Thilly N, Derrough T (2020). Human papillomavirus vaccination coverage, policies, and practical implementation across Europe. Vaccine.

[32] Perlman S, Wamai RG, Bain PA, Welty T, Welty E, Ogembo JG (2014). Knowledge and awareness of HPV vaccine and acceptability to vaccinate in sub-saharan Africa: a systematic review. PLoS ONE.

[33] Ezeanochie MC, Olagbuji BN (2014). Human papillomavirus vaccine: determinants of acceptability by mothers for adolescents in Nigeria. Afr J Reprod Health.

[34] Nogueira-Rodrigues A, Flores MG, Macedo AO (2022). HPV vaccination in Latin America: Coverage status, implementation challenges and strategies to overcome it. Front Oncol.

[35] Acampora A, Grossi A, Barbara A et al. Increasing HPV vaccination uptake among adolescents: a systematic review. Int J Environ Res Public Health 2020, 17(21). 10.3390/ijerph17217997 PMC7663345 33143171

[36] Jaspers L, Budiningsih S, Wolterbeek R, Henderson FC, Peters AA (2011). Parental acceptance of human papillomavirus (HPV) vaccination in Indonesia: a cross-sectional study. Vaccine.

[37] Boakye AE, Nair M, Abouelella DK et al. Trends in reasons for human papillomavirus vaccine hesitancy: 2010–2020. Pediatrics. 2023. 10.1542/peds.2022-060410 PMC10233736 37218460

